# Rhubarb enhances gastrointestinal motility via calcium-mediated intestinal acetylcholine release: a network pharmacology study

**DOI:** 10.1038/s41598-026-39372-z

**Published:** 2026-02-13

**Authors:** Zhiyong Wen, Weihua Liu, Jialing Li, Songling Tan, Jianbo Wen

**Affiliations:** Present Address: Gastroenterology Department, Pingxiang People’s Hospital, Pingxiang, 337000 China

**Keywords:** Acute pancreatitis, Rhubarb, Calcium ion pathway, Network pharmacology, Diseases, Gastroenterology, Pathogenesis, Drug development, Target validation

## Abstract

Rapid restoration of intestinal motility is crucial in managing acute pancreatitis (AP), particularly in severe cases. This study aims to investigate the potential of Chinese medicinal rhubarb to enhance bowel motility through modulation of calcium ion signaling in the gastrointestinal tract. A multi-omics approach integrating network pharmacology, KEGG pathway enrichment, and protein–protein interaction (PPI) analyses to identify rhubarb’s active components and target genes linked to AP. KEGG analysis highlighted key genes in the calcium ion pathway, facilitating the construction of a network pharmacology framework. C57BL/6 mice were single-blind randomly given saline or rhubarb gavage (0.2 mL/10 g, thrice daily for 2 days). Ileum tissues and serum were collected to measure key genes, acetylcholine (ACh), and acetylcholinesterase (AChE). Similarly, mild to moderate AP patients in Pingxiang People’s Hospital were single-blind randomly assigned to control or rhubarb groups, with serum analyzed for AChE levels. Multi-dimensional analyses identified ten active components in rhubarb that interact with 67 target proteins. Cross-referencing with AP-related targets identified 55 key regulatory genes, ten of which were significantly enriched in the calcium signaling pathway. Animal studies demonstrated that rhubarb significantly increased ACh in the mice ileum (*p* = 0.0023) and serum AChE levels (*p* < 0.0001). Q-PCR analysis indicated upregulation of nine calcium pathway-related genes. In a randomized single-blind trial with 10 AP patients, those receiving oral rhubarb had significantly more frequent bowel movements (*p* < 0.0001) and higher serum AChE levels (*p* < 0.0001) than controls. No adverse events occurred. Rhubarb increases diarrhea in mice by enhancing gastrointestinal motility, activating calcium ion signaling, and boosting ACh and AChE release. It also raises serum AChE levels and defecation frequency in AP patients. These effects may explain rhubarb’s therapeutic role in treating gastrointestinal motility disorders linked to AP.

Trial registration number: ITMCTR2025002044.

## Introduction

Acute pancreatitis (AP) is characterized by the premature activation of pancreatic enzymes triggered by various etiological factors, resulting in local and systemic inflammatory responses. Severe acute pancreatitis (SAP) can result in respiratory, circulatory, or renal organ failure persisting for more than 48 h^1^. Intestinal dysfunction is a common complication observed in patients with SAP^1^. Intestinal dysfunction in AP is contributed to by multiple factors, such as intestinal microcirculation disturbances, ischemia–reperfusion injury, release of inflammatory mediators, apoptosis of intestinal epithelial cells, and nutrient deficiencies, all of which ultimately influence disease prognosis^2^. Consequently, the early diagnosis and management of intestinal dysfunction are crucial for the comprehensive AP treatment^1–3^. In China, the use of rhubarb and its formulations to enhance gastrointestinal motility in AP has a long-standing tradition. Rhubarb (Emodin 6-methyl-1,3,8-trihydroxyanthraquinone) is derived from the dried, peeled roots and rhizomes of various perennial plants belonging to the Polygonaceae family and primarily contains anthraquinones (such as rhein, emodin, and sennosides A, B, C, and D) and tannins^4,5^. Previous studies have demonstrated that rhubarb improves gastrointestinal motility, reduces gastrointestinal contents, alleviates nausea, vomiting, and abdominal bloating in pancreatitis, shortens hospital stays, and lessens disease severity^6–8^. The typical dosage for improving gastrointestinal dysfunction ranges from 1.25 to 5 g/kg/d^9–11^. However, rhubarb contains up to 92 medicinal compounds with complex pharmacological mechanisms, and the specific active components and mechanisms responsible for enhancing gastrointestinal motility in AP remain unclear.

In the context of gastrointestinal motility, the electrical activity of smooth muscle cells within the digestive tract is currently understood to be associated with intracellular Ca^2+^ transport^12^. Gastrointestinal motility is regulated by sympathetic and parasympathetic nerves, gastrointestinal hormones, inflammatory mediators, and opioids. It is facilitated by modulation of calcium ion pathways to increase calcium influx, activating M receptors, and enhancing neurotransmitter release, such as ACh^13^. Early studies demonstrated that emodin-treated isolated guinea pig colonic smooth muscle cells exhibited enhanced contraction, linked to inhibition of potassium ion channel currents, increased intracellular calcium, and elevated ACh release^14^. Nevertheless, the specific genes, proteins, and molecular pathways through which rhubarb influences calcium ion channels remain to be elucidated. Therefore, this study hypothesizes that the effective components of rhubarb can modulate Ca^2+^ transport and augment ACh release to improve gastrointestinal motility.

## Results

### Active constituents of rhubarb and their corresponding target sites

**Fig. 1:**
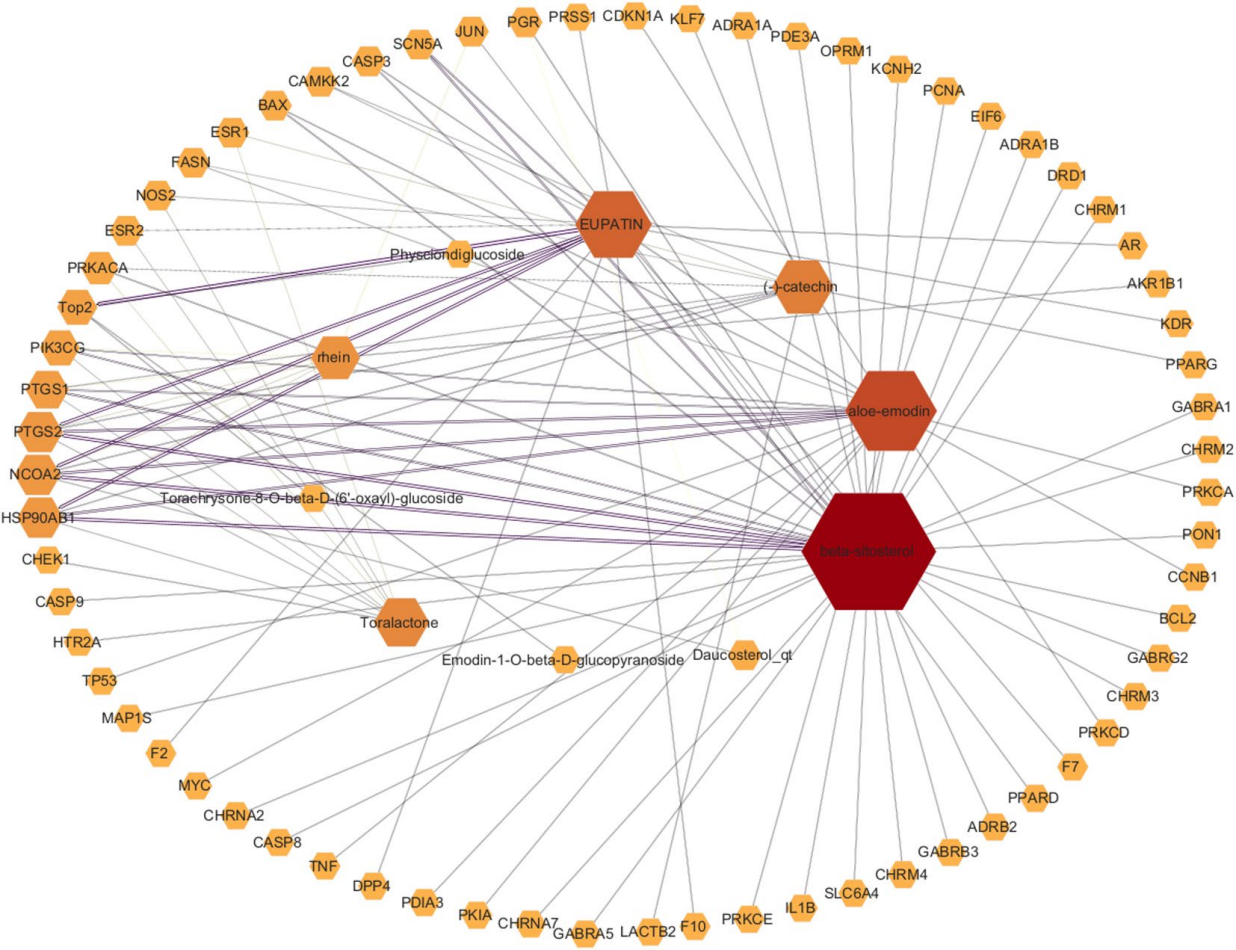
Active ingredients and target genes of rhubarb: The TCMSP identified 16 active compounds from rhubarb and 110 associated targets. Sixty-seven target genes were extracted from the Uniprot database, and Cytoscape software was used to create a weighted graph illustrating the relationships among the component nodes.

Using the TCMSP database, 16 active compounds in rhubarb were identified based on established screening criteria. Of these, ten compounds corresponded to 110 target proteins, which were subsequently mapped to human genes utilizing the UniProt database, yielding 104 targets. After removing duplicates, 67 unique target genes were obtained. The ingredient–target interactions were analyzed using Cytoscape software. As illustrated in Fig. 1, following weight mapping based on the number of component nodes (degree), it was determined that β-sitosterol, aloe-emodin, catechin, and geniposide were associated with a higher number of target genes, thereby identifying them as key active ingredients (Fig. 1).

### Enrichment of common target genes of AP and rhubarb within the gastrointestinal motility pathway

**Fig. 2:**
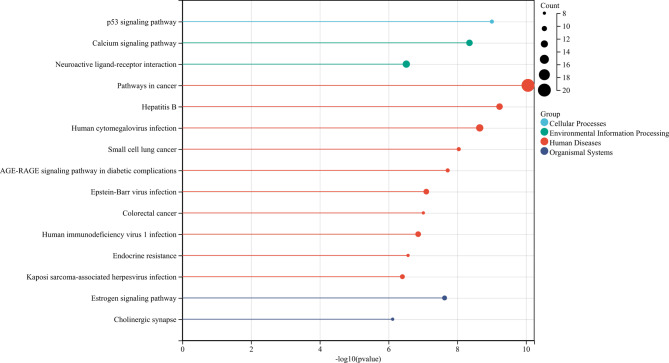
KEGG pathway map of 55 key genes: The 55 key genes were analyzed using R for KEGG pathway^16^ enrichment with the clusterProfiler package (version 3.14.3). A *p* value of < 0.05 and a false discovery rate (FDR) of < 0.25 were considered significant. The third pathway related to cellular processes was the neuroactive ligand-receptor interaction. The right y-axis represents the number of 55 genes in the target pathway.

From the disease databases CTD and GENECARDS, a total of 9456 disease target genes associated with AP were identified after duplicate removal. Comparative analysis with the target genes of rhubarb revealed 55 common genes, as illustrated in Fig. 2. The 55 key genes were analyzed using R for KEGG pathway enrichment with the clusterProfiler package (version 3.14.3)^15^, which identified the calcium ion transport pathway as the third-ranked pathway based on adjusted p-values, encompassing 10 genes from the 55 identified. The genes involved in this pathway include NOS2, DRD1, HTR2A, PRKCA, PRKACA, ADRB2, CHRM2, CHRM3, CHRNA7, and CHRM1.

### Construction of the network pharmacology diagram reveals β-sitosterol and other key active ingredients

**Fig. 3:**
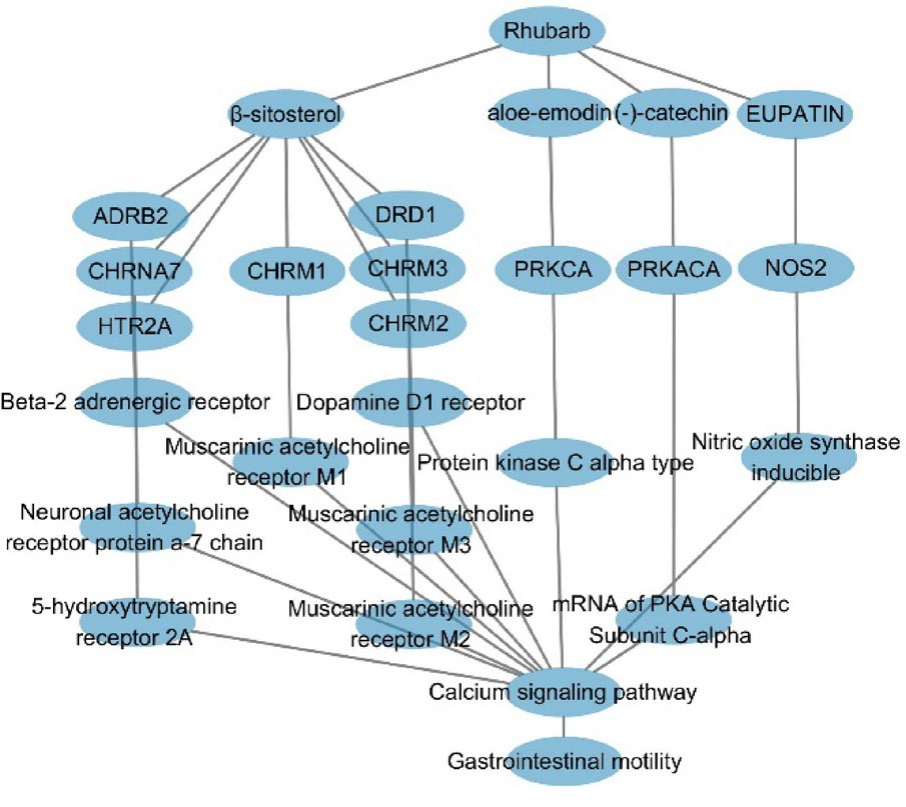
Network pharmacology of rhubarb active ingredients-targets-acute pancreatitis: Genes linked to the neural active ligand-receptor pathway will align with rhubarb’s active ingredients and drug targets. An Excel file will create a pharmacological network connecting these elements with pathways and diseases.

The gastrointestinal motility genes were correlated with the active compounds of rhubarb and their corresponding drug targets, leading to the creation of an attribute file for cytoscape software in Excel. This facilitated the construction of a network pharmacology diagram that delineates the interactions among rhubarb’s active ingredients, drug targets, pathways, and diseases, as depicted in the Fig. 3. Among the ten active compounds identified in rhubarb, β-sitosterol, aloe-emodin, catechin, and eupatin were found to regulate all target genes associated with gastrointestinal motility, including the ten genes mentioned earlier. Notably, β-sitosterol was observed to modulate the greatest number of drug targets, affecting the dopamine D1 receptor, serotonin 2A receptor, muscarinic acetylcholine receptors M1, M2, and M3, as well as the neuronal acetylcholine receptor protein α-7 chain and adrenergic receptor 2, among others.

### Construction of the target protein interaction network uncovers key target genes

**Fig. 4:**
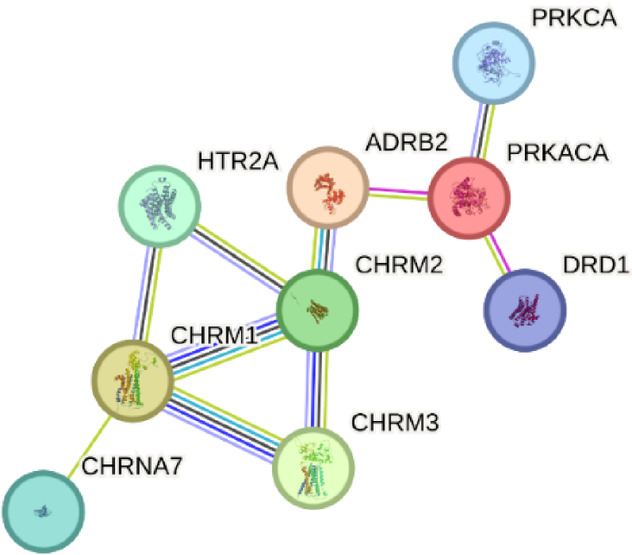
Interaction map of key gene PPI protein network: pathway genes were uploaded to the STRING database to create a protein–protein interaction (PPI) network map, analyzing relationships with over four target genes to identify key targets.

Genes associated with calcium ion transport and neuro-ligand receptor exchange pathways were analyzed using these STRING databases to construct a protein–protein interaction (PPI) network, as shown in the Fig. 4. By filtering for target genes with four or more interactions, we identified key target genes. The prominent target genes identified from the pathways included DRD1, HTR2A, PRKCA, PRKACA, ADRB2, CHRM1, CHRM2, CHRM3, and CHRNA7.

### Kyoto Encyclopedia of Genes and Genomes (KEGG) pathway analysis

The genes involved in regulating the calcium ion transport pathway were analyzed using the KEGG database to elucidate the upstream and downstream regulatory interactions among these genes. The analysis revealed that within the calcium ion transport pathway, target genes regulated by β-sitosterol—such as HTR2A, CHRM2, ADRA1A, ADRA1B, CHRM1, CHRM3, ADRB2, and DRD1—are associated with the modulation of G protein-coupled receptors. These receptors influence neurotransmitter binding, thereby affecting the release of calcium ions from the endoplasmic reticulum and maintaining intracellular calcium ion homeostasis, which is critical for the proper functioning of gastrointestinal smooth muscle.

### Validation of ACh and AChE analysis in animal experiments

**Fig. 5:**
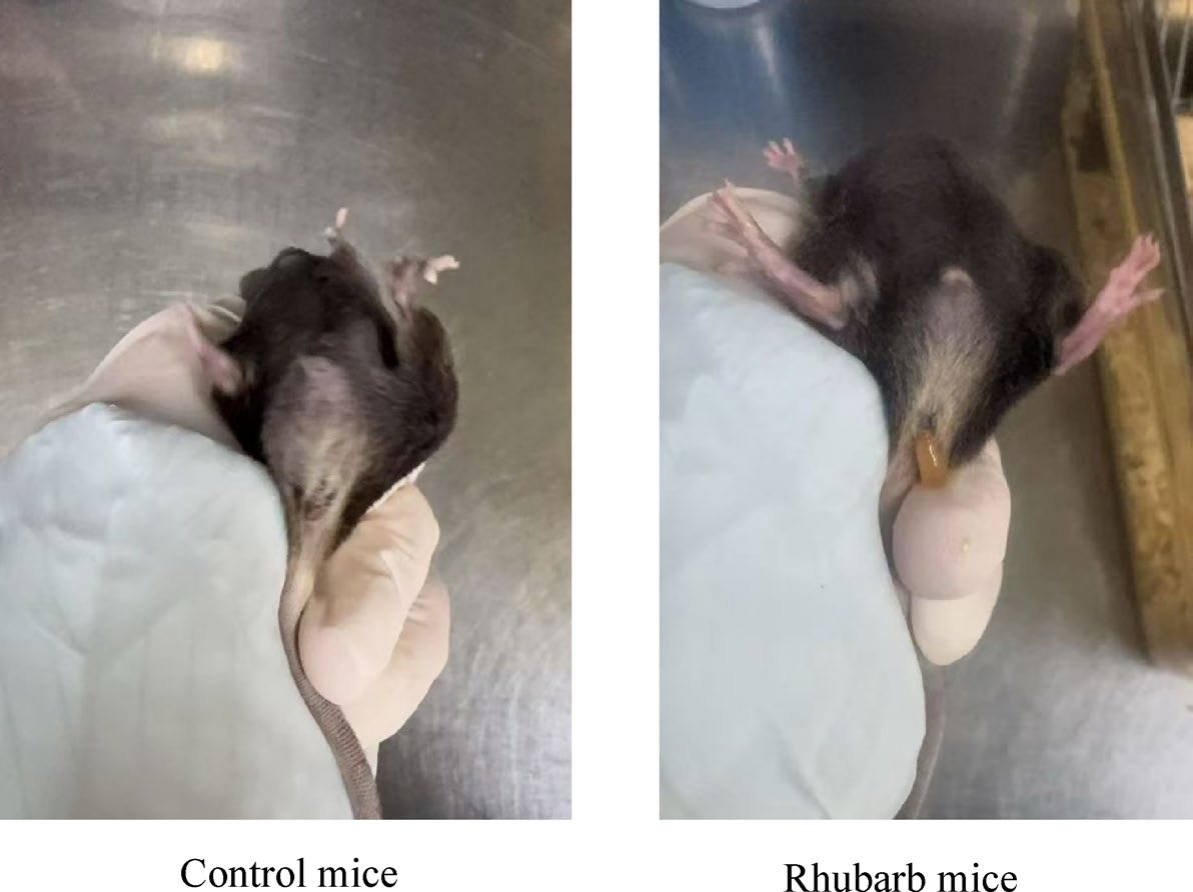
Mice given rhubarb: C57BL6 mice were gavaged with rhubarb, and 2 days later, they showed diarrhea symptoms.

**Fig. 6:**
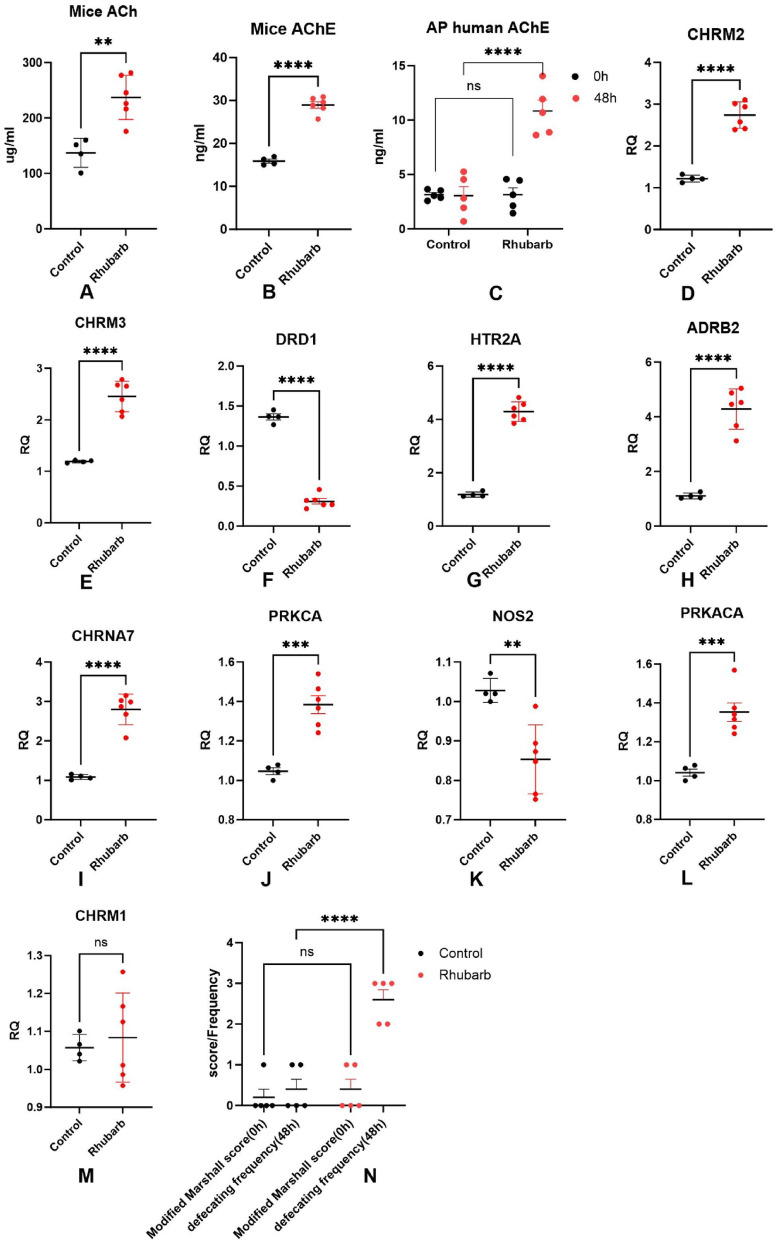
ACh(A) and q-PCR(D-M) results of key genes in the terminal ileum of mice. Serum AChE levels were measured separately in mice (Fig. 6B) and human patients with AP (Fig. 6C). Figure 6N illustrates disease severity at enrollment in acute pancreatitis patients and bowel movement frequency during the 48 h after rhubarb administration. Asterisks indicate statistical significance: *P < 0.05, **P < 0.01, ***P < 0.001, ****P < 0.0001; ns = not significant.

C57BL/6 mice were administered rhubarb via gavage following the established experimental protocol. Two days after administration, mice in the experimental group exhibited symptoms of diarrhea (Fig. 5), indicating that rhubarb enhances gastrointestinal motility and induces diarrhea in this model. ACh levels in the terminal ileum were measured in both experimental and control groups. As shown in Fig. 6A, the experimental group demonstrated a statistically significant increase in ACh concentration within the terminal ileum. Furthermore, serum AChE activity was evaluated (Fig. 6B), revealing a marked increase in the experimental group compared to controls.

The key genes identified were assessed in the terminal ileum of mice intestines. The results demonstrated statistically significant differences in the expression levels of CHRM2, CHRM3, DRD1, HTR2A, ADRB2, CHRNA7, PRKCA, NOS2, and PRKACA (Fig. 6D–L), while the expression of CHRM1(Fig. 6M) did not show a significant difference.

### Validation of AChE analysis in clinical experiments

Ten patients with acute pancreatitis were recruited to examine rhubarb’s effect on serum AChE; all completed the study without adverse events. No significant differences in gender, age, or disease severity were found between groups (gender and age data not shown, Fig. 6N). Following 48 h of rhubarb administration, the treatment group exhibited elevated serum AChE levels relative to the control group. (Fig. 6C). Additionally, the treatment group had a significantly higher frequency of bowel movements during this period (Fig. 6N).

## Discussion

AP is a common gastrointestinal disorder, with approximately 15–20% of affected patients progressing to severe acute pancreatitis. The early phase of severe pancreatitis can induce systemic inflammatory response syndrome and multiple organ dysfunction syndrome (MODS), primarily due to the substantial release of inflammatory cytokines. In contrast, the later stages of the disease may result in intestinal dysfunction and pancreatic necrosis^1^. Research indicates that 59% of patients with AP experience damage to the intestinal barrier, characterized by increased intestinal mucosal permeability. This impairment can result in bacterial translocation, pancreatic tissue necrosis, and subsequent infections, ultimately leading to MODS^2,3^. The use of rhubarb in the treatment of pancreatitis has been validated in numerous clinical trials. This study investigates the potential of rhubarb, a traditional Chinese medicine, to enhance bowel motility through the modulation of calcium ion signaling in the gastrointestinal tract.

This study employed network pharmacology to identify ten bioactive compounds in rhubarb associated with acute pancreatitis, alongside fifty-five related target genes. KEGG pathway analysis indicated that ten of these target genes are involved in the calcium ion signaling pathway and acetylcholine release, both of which play critical roles in regulating gastrointestinal motility. In animal studies, rhubarb administration under physiological conditions induced diarrhea in mice, enhanced ACh release in the ileum, and upregulated the expression of nine specific target genes, while simultaneously increasing AChE levels in mouse serum. Previous research has shown that rhubarb promotes intestinal motility, increases fecal water content in mice, and stimulates ACh release from isolated intestinal tissues, effects attributed to rhubarb’s stimulation of neurosecretion from mast cells^17^. Clinical studies further revealed that rhubarb elevates serum AChE concentrations in patients with AP. Although alterations in intestinal ACh levels and target gene expression were not evaluated in the context of acute pancreatitis, these findings provide preliminary evidence suggesting that the therapeutic mechanism of rhubarb—supported by clinical practice and treatment guidelines—for managing gastrointestinal motility disorders in acute pancreatitis may involve activation of calcium ion signaling pathways to promote acetylcholine release. The subsequent section examines the relationships among target genes, gastrointestinal motility, calcium signaling, and acetylcholine regulation.

Neuronal nicotinic acetylcholine receptors (nAChRs) are cation-selective, calcium-permeable ion channels. The α7 nAChR subtype exhibits the highest calcium-to-sodium permeability ratio (PCa/PNa), indicating its critical role in modulating neurotransmitter release, gene expression, neuroprotection, and neurotoxicity^18,19^. Our animal model studies have demonstrated an upregulation of the CHRNA7 gene, indicating that rhubarb enhances the activation of CHRNA7 receptors. This activation subsequently affects G protein-coupled receptor-mediated signaling pathways, elevating intracellular calcium ion concentrations and promoting acetylcholine synthesis. Previous research supports the notion that CHRNA7 mediates calcium ion influx to regulate acetylcholine release during neurotransmitter transmission^18^.

The DRD gene encodes the D1 subtype of dopamine receptors, which belong to the family of G protein-coupled receptors. Immunoblotting has confirmed the presence of D1, D2, and D5 receptor proteins from the stomach to the distal colon. In mice with knockout mutations of D2, D3, or both receptors, gastrointestinal transit time (TGTT) and colonic transit time (CTT) were significantly reduced, indicating that dopamine receptor activation plays an inhibitory role in gastrointestinal motility^20^. Studies examining distal colonic motility have demonstrated that dopamine receptor D1 mediates the inhibitory effects of dopamine on this motility^20^. Our results also reveal its decrease in DRD1 expression following rhubarb administration, suggesting a potential role in gastrointestinal motility. The underlying mechanism may involve dopamine’s capacity to block calcium channel currents (ICA), thereby reducing calcium influx^21^, while rhubarb’s inhibition of DRD1 receptor expression may counteract dopamine’s inhibitory effects on calcium channels.

Muscarinic acetylcholine receptors (mAChRs) belong to the G protein-coupled receptor superfamily and comprise five identified subtypes (M1–M5)^22,23^. The odd-numbered muscarinic receptors (M1, M3, M5) signal through Gq/11, activating PLC to hydrolyze PIP2 into inositol trisphosphate (IP3) and diacylglycerol. IP3 induces the release of Ca^2^⁺, while diacylglycerol activates protein kinase C (PKC). Conversely, the even-numbered receptors (M2, M4) couple to Gi/o, inhibiting adenylyl cyclase and promoting K^+^ channel activation, leading to membrane hyperpolarization^23,24^. In our study, no significant differences were observed in the expression of the CHRM1 neuroreceptor-ligand pathway within mouse intestinal tissue, which may be attributable to variations in receptor distribution. Previous studies have demonstrated that 7,8-dihydroxyflavone (7,8-DHF), a synthetic agonist of the tyrosine kinase receptor B (TrkB), enhances gastric motility in rats by activating M2 receptors and increases colonic motility via the TrkB/Akt/M3 pathway, thereby alleviating constipation^25^. Antagonism of CHRM3 reduces ACh, leading to smooth muscle contraction in equine tissues. Notably, M2 receptors modulate contraction via neurohormonal mechanisms rather than direct cholinergic activity^26^. Activation of CHRM3 enhances motility in the small intestine but not in the colon of mice, indicating site-specific effects13. CHRM2 and CHRM3 do not act alone; they form a complex activating TRPC4 channels to promote visceral smooth muscle contraction^27^. Reduced expression of both receptors in megacolon impairs ACh transmission and intestinal motility^28^. Both receptors are involved in calcium signaling pathways and exhibit co-expression and simultaneous activation, suggesting that rhubarb may enhance intestinal motility by upregulating CHRM2/CHRM3 or by increasing intracellular calcium to influence acetylcholine release^12^.

HTR2A and ADRB2 are G protein-coupled receptors. This study observed upregulated expression of both genes within ion transport pathways. The 5-HT2A receptor, a principal serotonin (5-HT) subtype, mediates calcium release through the Gq–PLC–IP3–Ca^2^⁺ pathway, promoting smooth muscle contraction and modulating gastrointestinal motility^29,30^. Additionally, paracrine 5-HT acts on enteric nervous system (ENS) 5-HT2A receptors, activating cholinergic neurons and enhancing acetylcholine release to facilitate peristalsis^31^. In diarrhea-predominant irritable bowel syndrome (IBS), treatments such as Tongxie Xiaoyao San^32^ and Lactobacillus plantarum^33^ alleviated symptoms by reducing colonic 5-HT, further implicating 5-HT receptors in promoting motility. ADRB2 regulates calcium release via the Gs–cAMP–PKC pathway. Activation of ADRB2 by albuterol enhances skeletal muscle movement by increasing sarcoplasmic Ca^2^⁺ release^34^. Earlier gastrointestinal studies reported that ADRB2 activation delays gastric emptying in vivo^35^ and inhibits isolated ileum contractions^36^, suggesting its inhibitory role via neural pathways. Additional research indicated that diprophylline modulates contraction force in rat jejunum depending on the muscle’s contraction state, reflecting bidirectional regulation of gastrointestinal function stability^37^. Similarly, daidzein had been reported to reduce hypercontraction of isolated small intestinal smooth muscle^38^. Based on these findings, we hypothesize that rhubarb administration activates ileal ADRB2 receptors to regulate excessive gastrointestinal motility; however, further investigation is required to elucidate the underlying mechanisms.

NOS2 encodes inducible nitric oxide synthase (iNOS), an enzyme responsible for producing substantial quantities of nitric oxide (NO). Excessive NO inhibits the excitability of enteric cholinergic neurons and the release of ACh through activation of the soluble guanylate cyclase (sGC)-cyclic guanosine monophosphate (cGMP)-protein kinase G (PKG) signaling pathway. It also disrupts neuron and interstitial cells of Cajal (ICC) networks and counteracts ACh-induced smooth muscle contraction. Protein kinase G 1 (PKG1) phosphorylates inositol 1,4,5-trisphosphate receptor-associated cGMP kinase substrate (IRAG), thereby inhibiting IP₃ receptor-mediated calcium release and lowering calcium levels^39^. Our study found reduced NOS2 expression in the terminal ileum of mice, indicating that rhubarb may suppress intestinal NO production, elevate intracellular calcium ion concentrations in intestinal cells, and promote smooth muscle motility.

PRKCA encodes PKCα, a classic protein kinase C subtype. When calcium ions bind to PKCα’s C2 domain, it moves to the plasma membrane, where it interacts with phosphatidylserine (PS) and diacylglycerol (DAG) to activate. Activated PKCα phosphorylates L-type voltage-gated calcium channels (LTCC), increasing calcium influx^40^. PRKACA encodes the catalytic subunit α of protein kinase A (PKA), which phosphorylates substrates to produce various biological effects. In intestinal smooth muscle cells, PKA enhances calcium influx and storage, promoting vesicle fusion and acetylcholine release from nerve terminals^41^. Our study observed upregulated expression of PRKCA and PRKACA in the mouse terminal ileum, indicating that rhubarb may increase intracellular calcium ion concentrations, stimulate acetylcholine release, and consequently improve gastrointestinal motility.

In conclusion, the bioactive compounds in rhubarb—β-sitosterol, aloe-emodin, catechin, and eupatin—upregulated nine genes associated with the calcium ion signaling pathway in mice. Rhubarb enhances intestinal motility, increases bowel movement frequency, and softens stools in both normal mice and AP patients. It also increased ACh release in the intestines and serum AChE activity in both mice and acute pancreatitis patients. However, the study lacked protein-level validation using a recognized AP animal model and direct calcium flux measurement, limiting the conclusiveness of its results. Despite these limitations, using network pharmacology to study rhubarb’s effects in animals and clinical cases provides preliminary pharmacological support for its therapeutic use.

## Materials and methods

### Tools

The following databases and software were used: TCMSP (https://www.tcmsp-e.com/index.php), UniProt (https://www.uniprot.org/), Comparative Toxicogenomics Database (CTD, https://ctdbase.org/), DisGeNET(https://disgenet.com/), STRING (https://cn.string-db.org/), Kyoto Encyclopedia of Genes and Genomes (KEGG; http://www.genome.jp/kegg/) databases; Cytoscape (v3.10.2) and R language (v4.1.2).

### Active components and target genes of rhubarb

Active components of rhubarb were retrieved from TCMSP using “rhubarb” as the keyword, with screening criteria set as oral bioavailability (OB) ≥ 30% and drug-likeness (DL) ≥ 0.18. Target proteins were predicted using TCMSP, then converted to standard gene names via UniProt, with non-gene entries and non-human targets excluded.

### Common target genes for acute pancreatitis and rhubarb

AP-related targets were collected from CTD and DisGeNET using “acute pancreatitis” as the keyword. After merging and removing duplicates, common targets between rhubarb and AP were identified.

### Construction of a network pharmacology diagram for rhubarb in treating pancreatitis

The active components and target genes of rhubarb were imported into Cytoscape software for analysis and topological assessment to construct a network illustrating the key active components and target genes involved in the treatment of AP.

### KEGG pathway enrichment analysis

Enrichment analysis of common targets was performed using the R package clusterProfiler (v3.14.3). Results were used to identify significantly enriched pathways, particularly those related to calcium ion signaling.

### PPI network construction

Common targets were imported into STRING with species limited to “Homo sapiens”. disconnected nodes were hidden, and a minimum interaction confidence score of 0.4 was applied. The PPI network was generated under default parameters.

### Network pharmacology model integrating calcium pathways

Active components of rhubarb and calcium pathway-related target genes were analyzed in Cytoscape to construct a comprehensive network illustrating component–target–pathway interactions in the context of AP.

### Validated experimental

#### Animal and subject inclusion

Ten SPF C57BL/6 mice (Hunan Sileke Jingda Experimental Animal Co., Ltd.; 4–6 weeks old, 15–20 g) were acclimatized for 1 week and randomly assigned to a control group (n = 4) or an experimental group (n = 6). This study recruited 10 AP patients treated at the Department of Gastroenterology, Pingxiang People’s Hospital, from September 2024 to May 2025. Inclusion criteria include age 18–65 years, diagnosis confirmed by Modified Marshall score, ability to tolerate oral intake, no cognitive or communication impairments, and informed consent. Exclusion criteria include malignancy, cardiovascular or cerebrovascular disease, severe residual dysfunction, history of constipation or gastrointestinal surgery, pregnancy, emergency surgery, blood purification, or other investigator-judged contraindications. Patients requiring emergency surgery, experiencing condition worsening, or deemed necessary for withdrawal by investigators will be removed early.

#### Materials and instruments

Rhubarb powder (Gansu Tianxintang Pharmaceutical Co., Ltd., batch no. 2411006); ELISA kits for mouse ACh, mouse AChE, and human AChE (Shanghai Enzyme-linked Biotechnology Co., Ltd., cat. no. ML401805, ML037240, ML064256); real-time PCR system (ABI 7900); UV spectrophotometer (Shanghai Unico, UV-2355).

#### Experimental procedures

Mice in the experimental group received a rhubarb decoction (prepared by Pingxiang People’s Hospital TCM Department) made by dissolving 10 g raw rhubarb powder in 100 mL purified water at 70 °C, soaked for 10 min, cooled, and mixed thoroughly. Administration was by gavage at 0.2 mL/10 g body weight. The control group received an equal volume of saline. Treatments were given three times daily for 2 days until diarrhea occurred. Mice were euthanized by gradually increasing exposure to 100% CO_2_ at a fill rate of 30% of the chamber volume per minute, followed by confirmation of death via cervical dislocation. Terminal ileum samples were collected for gene expression and ACh measurement. Ethical approval was granted by the Ethics Committee of Pingxiang People’s Hospital (No. 2023D001-HS02). All procedures complied with relevant guidelines and regulations. The study design, animal experiments, statistical analysis, and data reporting adhered to ARRIVE guidelines.

This single-center, single-blind, randomized controlled trial assigned subjects to control or experimental groups in a 1:1 ratio using a web-based computer-generated randomization system. Both groups received standard care per the AP Diagnosis and Treatment Guidelines (2021). The experimental group was given 2 mL/kg rhubarb solution, while the control group received normal saline, three times daily for 2 days. Researchers recorded daily bowel movement frequency, volume, and stool characteristics. Rhubarb was discontinued if bowel movements exceeded three times per day, stool volume surpassed 200 g, or stool water content exceeded 85%. Serum samples were collected before and 48 h after treatment for AChE assay. The Ethics Committee of Pingxiang People’s Hospital approved the trial (No. SW-2023Z099-HS02). The study adhered to the Declaration of Helsinki and was registered with the International Traditional Medicine Clinical Trial Registry (ITMCTR2025002044; URL: https://itmctr.ccebtcm.org.cn. Registration date: October 28, 2025). Informed consent was obtained from all participants.

#### Detection methods

ACh and AChE levels in ileal tissue (mice) and serum (human) were measured using ELISA kits according to manufacturer instructions. Absorbance was read at 450 nm using a microplate reader, and concentrations were calculated via standard curve. qPCR was performed to assess mRNA expression of NOS2, DRD1, HTR2A, PRKCA, PRKACA, ADRB2, CHRM2, CHRM3, CHRNA7, and CHRM1 in mouse ileum, following MIQE guidelines. Each 10 μL reaction contained 10 ng cDNA, 200 nM primers, and SYBR Green Master Mix. Cycling conditions: 95 °C for 10 min; 40 cycles of 95 °C for 15 s and 60 °C for 30 s. Melt curve analysis confirmed specificity. β-actin was used as reference. Primers were designed via PrimerBank and synthesized by Sangon Biotech (Shanghai); sequences are listed in Table 1.

**Table 1:** q-PCR primer information.

Gene name	Primer sequence5′–3′	Gene name	Primer sequence5′–3′
F2-F	CCGAAAGGGCAACCTAGAGC	CHRM3-F	CCTCGCCTTTGTTTCCCAAC
F2-R	GGCCCAGAACACGTCTGTG	CHRM3-R	TTGAGGAGAAATTCCCAGAGGT
DRD-F	CACGGCATCCATCCTTAACCT	CHRM4-F	ATGGCGAACTTCACACCTGTC
DRD-R	TGCCTTCGGAGTCATCTTCCT	CHRM4-R	CTGTCGCAATGAACACCATCT
HTR2A-F	TAATGCAATTAGGTGACGACTCG	CHRNA7-F	AGTTTTAACCACCAACATTTGGC
HTR2A-R	GAGGCTTCGGAAGTGTTAGCA	CHRNA7-R	TTTTCACTCCGGGGTACTCAG
ADRB2-F	ATGTCGGTTATCGTCCTGGC	GABRA1-F	TGAGCACACTGTCGGGAAGA
ADRB2-R	GGTTTGTAGTCGCTCGAACTTG	GABRA1-R	CAGCAGTCGGTCCAAAATTCT
OPRM1-F	TCCGACTCATGTTGAAAAACCC	GABRG2-F	GCTCTACCCAGGCTTCACAAG
OPRM1-R	CCTTCCCCGGATTCCTGTCT	GABRG2-R	CCAGCAGGTTGTTTAAGATGACA
CHRM1-F	CCTGAGCTACCGAGCCAAG	GABRB3-F	AAGCTGTTGAAAGGCTACGAC
CHRM1-R	CCCAACTAGGTATTGCCAGAAG	GABRB3-R	ACTCGATTGTCAAGCGTGAGG
CHRM2-F	TGGTTTGGCTATTACCAGTCCT	β-actin-F	GTGACGTTGACATCCGTAAAGA
CHRM2-R	CTGAAGGTGGCGGTTGACTT	β-actin-R	GCCGGACTCATCGTACTCC

### Statistical methods

Statistical analyses were performed using Prism 9.0 software for data processing, evaluation, and visualization. Results are presented as mean ± standard deviation (x ± s). Comparisons between two groups employed t-tests and two-way ANOVA, with significance set at *P* < 0.05. Missing data will be addressed using multiple imputation. For small samples, pre-specified sensitivity analyses will assess the robustness of conclusions.

## Data Availability

The data is available from the corresponding author upon reasonable request.
